# Supplementary material for: Transparent origami glass
Source: Nat Commun. 2021 Jul 12;12:4261. doi: 10.1038/s41467-021-24559-x (PMC8275568; doi:10.1038/s41467-021-24559-x)
Supplement: Supplementary file 1 — supplementary informations [file 41467_2021_24559_MOESM1_ESM.pdf]

Supplementary Materials

Transparent Origami Glass

Yang Xu[1], *Ye Li*[1], *Ning Zheng*[1*], *Qian Zhao*[1,2], *Tao Xie*[1,2*]

[1]State Key Laboratory of Chemical Engineering, College of Chemical and Biological Engineering, Zhejiang University, 38 Zheda Road, Hangzhou, 310027, P. R. China
[2]ZJU-Hangzhou Global Scientific and Technological Innovation Center, Hangzhou, 311215, P. R. China

*Correspondence and requests for materials should be addressed to N.Z. (zhengning@zju.edu.cn) and T.X. (taoxie@zju.edu.cn)

Supplementary figures

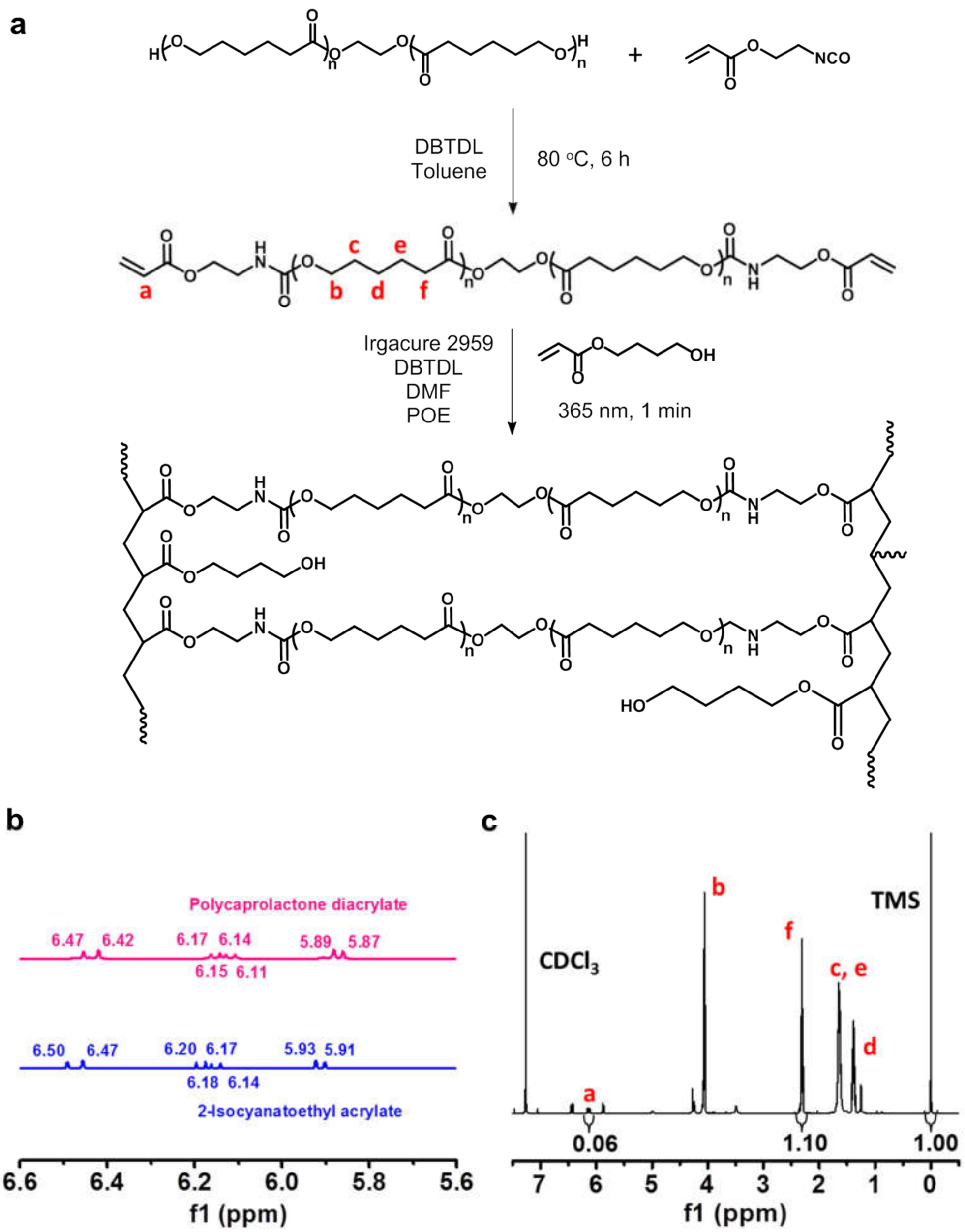

Supplementary Figure 1. **a**, Synthetic scheme for the dynamic polymer matrix. **b**. ^{1}H-NMR peak shift (acrylate) due to the conversion from 2-isocyanatoethyl acrylate to polycaprolactone diacrylate. **c**, ^{1}H-NMR spectrum of the synthesized polycaprolactone diacrylate. ^{1}H-NMR analysis was conducted using an Avance III500 spectrometer by Braker. The conversion of the polycaprolactone diol to polycaprolactone diacrylate is 90.5%, calculated from the equation $\psi = A_a N / A_f \times 100\%$, with A_a, A_f, and N being the area of the defined peak a, the area of the defined peak f, and the number of the repeating units in polycaprolactone, respectively. DBTDL, DMF, and POE stand for dibutyltin dilaurate, N,N-dimethylformamide, and phenoxyethanol, respectively.

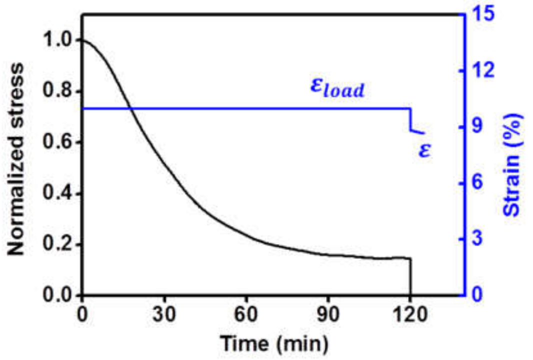

Supplementary Figure 2. The stress relaxation and strain curves of OH30 at 130 °C. The absolute stress at time zero was 0.18 MPa.

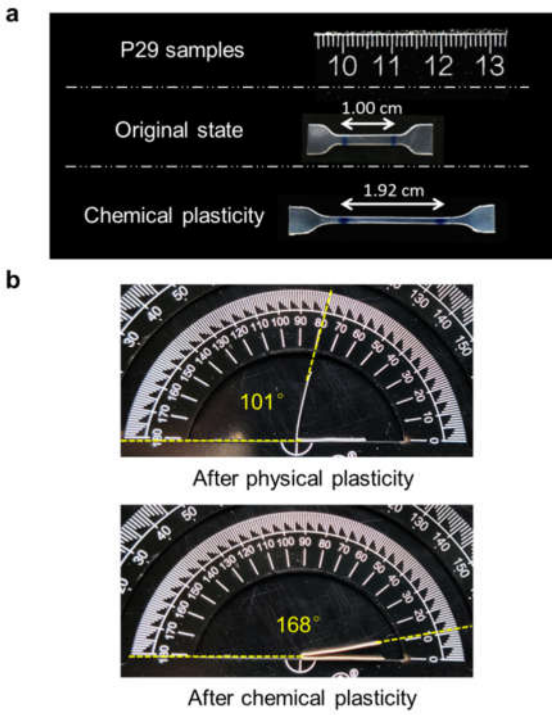

Supplementary Figure 3. The shape retention of the composite P29. **a** The tensile shape retention of composite P29 after chemical plasticity. **b** The bending shape retention of composite P29 after physical plasticity and chemical plasticity, respectively.

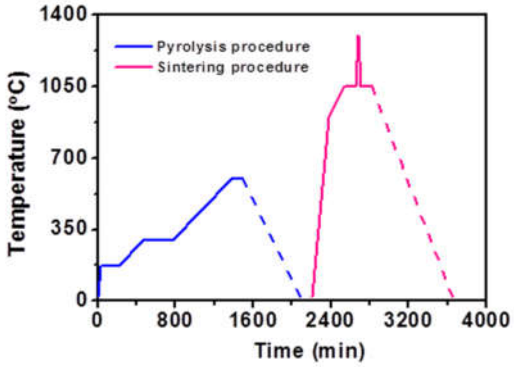

Supplementary Figure 4. The heating procedures of pyrolysis and sintering. The dashed lines denote natural furnace cooling and the temperature is not monitored. During the sintering step, the heating from 1050 °C to 1300 °C was designed to be rapid in order to utilize the kinetic difference between the surface migration and the grain growth to promote transparency of the resulting glass. The pyrolysis was conducted in the air in order to promote oxidation to avoid carbonization of the polymer. By comparison, sintering was performed under vacuum to ensure densification key to the transparency of the final glass.

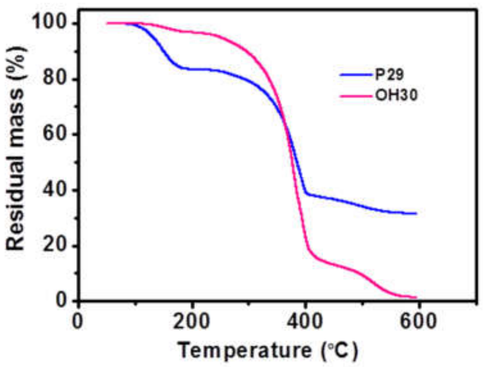

Supplementary Figure 5. TGA curves of the OH30 polymer matrix and the P29 composite. The measurement was conducted with a TA Q500 thermogravimetric analyzer. Heating rate: 10 °C/min.

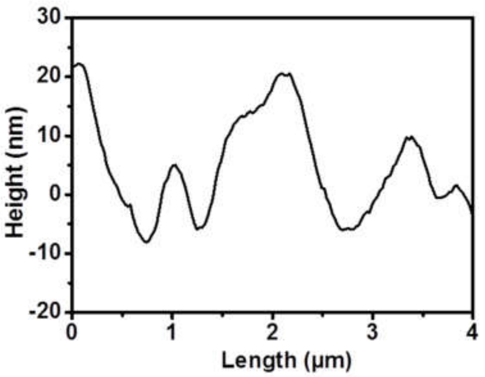

Supplementary Figure 6. Surface profile of the sintered glass obtained by atomic force microscopy measurement. The surface roughness R_a, defined as the arithmetical mean of the absolute value of the deviation of the surface profile, is within 17 nm.

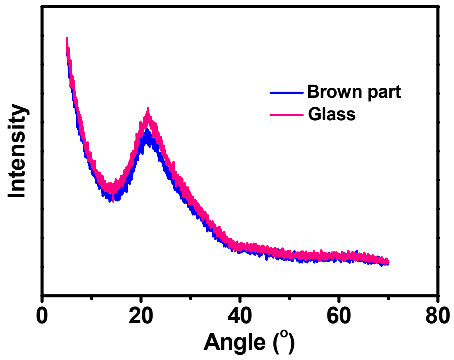

Supplementary Figure 7. The XRD analyses of the brown part and sintered glass. The broad diffraction peak indicates the amorphous nature of the sintered glass.

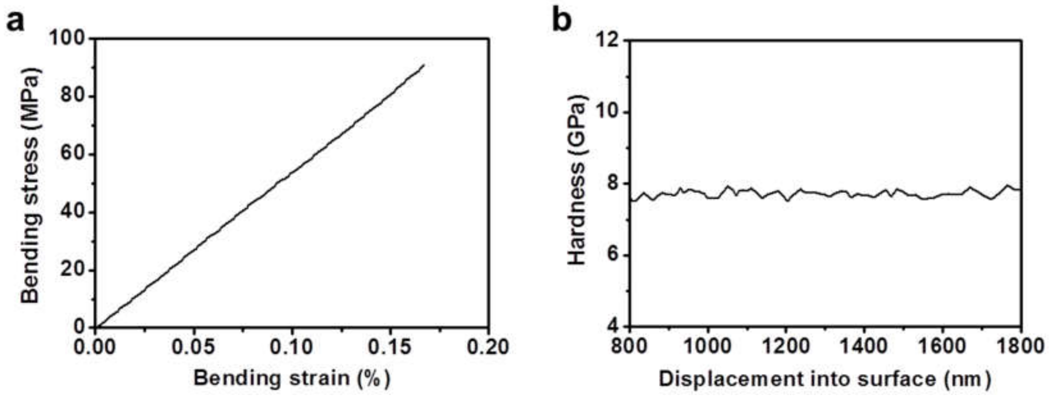

Supplementary Figure 8. The mechanical curves of the obtained glass. **a** A representative bending stress-strain curve. **b** A representative hardness curve of the obtained glass. The bending test was conducted with an Instron 5944 instrument at a testing speed of 10 μm/s at room temperature. The hardness was measured with an Aglient G200 nanoindenter at room temperature using a continuous stiffness measurement technique (the testing frequency is 45 Hz and the testing depth is 2000 nm).
